# From incomer to insider: The development of the TRANSPEC model – A systematic review of the factors influencing the effective rapid and early career TRANsition to a nursing SPECiality in differing contexts of practice

**DOI:** 10.1371/journal.pone.0216121

**Published:** 2019-05-01

**Authors:** Desley Hegney, Diane Chamberlain, Clare Harvey, Agnieszka Sobolewska, Bruce Knight, Anne Garrahy

**Affiliations:** 1 Research Division, Central Queensland University, Brisbane, Queensland, Australia, School of Nursing, The University of Adelaide, Adelaide, Australia; 2 College of Nursing & Health Sciences, Flinders University, Adelaide, South Australia, Australia; 3 School of Nursing, Midwifery and Social Science, Central Queensland University, Townsville, Queensland, Australia; 4 School of Nursing, Midwifery and Social Science, Central Queensland University, Brisbane, Queensland, Australia; 5 School of Education and the Arts, Central Queensland University, Townsville, Queensland, Australia; 6 A/Director, Employment Relations, Queensland Health, Employment Relations Unit, Human Resources Branch, Corporate Services Division, Brisbane, Queensland, Australia; University of Texas at Austin, UNITED STATES

## Abstract

**Objective:**

Shortages in the speciality nursing workforce, both nationally and internationally are driving the need for the development of an evidence-based model to inform recruitment and retention into speciality nursing practice. This study aimed to identify the factors influencing rapid and early career transition into speciality nursing practice.

**Methods:**

A comprehensive systematic review of the literature was undertaken using a convergent qualitative synthesis design where results from qualitative, quantitative and mixed methods studies were transformed into qualitative findings. Databases included CINAHL, Medline, Scopus and PsycINFO. Search terms were: nurse, early career, rapid career, transition, specialty, and Medical Subject Heading terms included: professional development and educational, nursing, and continuing. Using validated tools, papers were independently assessed by a minimum of two reviewers.

**Results:**

Twenty-three research articles were included. There were no randomized control trials. Through thematic analysis and matrix mapping of the results, the TRANSPEC model was developed. The model outlines three phases of transition: pre-entry, incomer and insider. There has been little focus on pre-entry with programs being designed at the incomer and insider phases. Impacting on these phases are three concepts: the self (professional and personal), the transition processes (informal and formal) and a sense of belonging. The overarching theme influencing the phases and concepts is the context of practice. Enablers and inhibitors influence successful transition and therefore impact on recruitment and retention. Each nurse’s transition is influenced by time.

**Conclusions:**

For successful transition, the enablers and inhibitors impacting on the three concepts, phases and the context of practice need to be considered when developing any program. It is apparent that while previous studies have focused on the transition processes, such as curricula, the development of the self and a sense of belonging are also essential to successful transition. Further studies should include the pre-entry phase.

## Introduction

The need to re-examine the numbers and the types of nurses, and their role within the health workforce arises due to many factors including population drivers; economic considerations such as increased costs of medical advances and the use of technology to provide services; and problems with health workforce supply and mal-distribution [1]. Population drivers include an increased demand on health services driven by the increased life expectancy of populations, particularly in developed countries, alongside the rising number of people living with chronic conditions who require more complex and long term treatment [2–4]. In Australia, a major driver of the change in health service delivery has been the 50% of the population with at least one chronic condition, as 40% of all preventable hospital admissions are related to chronic conditions [5]. Servicing the need for this population alone has driven up the cost of provision of services. Described as a “growing health crisis” [6], the increased cost for treatment has resulted in an increased acuity of patients in the acute care system, with a flow on effect of earlier discharge in an attempt to reduce the length of hospitals stays. The flow on effect has meant that patients who are sicker are being cared for in their homes by multidisciplinary teams from numerous specialty areas and service providers [6]. The cost of medical devices, particularly the increase in availability and use of technology for diagnosis and treatment [1] and Tele-health provision, particularly to populations in rural and remote areas, has also driven the cost of delivery [2]. These changes have resulted in a need to re-examine traditional ways that health professionals provide services within hospitals and the community.

Complicating this role redefinition, has been workforce supply and mal-distribution. Similar to its population, Australia has an ageing health workforce, many of which have reduced their working hours as they prepare for retirement [5]. The preference of many health professionals, particularly medical practitioners, to work in metropolitan areas, has resulted in a mal-distribution of that workforce. While this mal-distribution has been the focus of the Australian Government for many years, it is now increasingly evident that, while the mal-distribution of medical practitioners may continue, driving a shortage in rural and remote areas, there is now a growing overall shortage of nurses in all geographical areas as well as in specific areas of speciality nursing practice [5, 7]. The overall response of the Australian government to address the mal-distribution and shortage has been to increase supply [8]. Similar shortages have occurred in other countries such as the United States of America and the United Kingdom, for several decades [9, 10]. However, Buchan and Aiken [11] also suggest that the reasons for the shortages are complex and not limited to actual numbers entering the profession.

In Australia there are three levels of nurses. Two levels are regulated by the National Body: Australian Health Practitioner Agency (AHPRA),–the registered nurse (RN) and the enrolled nurse (EN). The RNs complete a university degree (Master or Bachelor), while the ENs mostly graduate with a diploma obtained from a technical college. The third level are unregulated care providers known as assistants in nursing (AIN) or Personal Care Workers (PCWs), who may or may not have certificate qualifications. The three levels of nurses work across all care settings, with the registered nurse held responsible for the care that is delegated to ENs/AINs. There are also nurse practitioners (NP) who were introduced to the workforce in 2001. NPs are experienced RNs who work as advanced clinicians following the completion of a specialised master’s degree. NPs are endorsed through AHPRA, have limited prescribing rights and manage their own caseload.

In Australia, it is predicted that there will be a shortfall of 85,000 nurses by 2025 mostly due to increased work intensification coupled with recruitment and retention issues [2]. The shortages have been noted in all areas of nursing, and in particular at the RN level, in specialities such as critical care, neonatal, aged care, emergency nursing, rural and remote areas and mental health nursing [4, 12, 13]. Internationally, the World Health Organization has predicted a global shortage of 7.6 million by 2030 [14].The focus on supply has resulted in more newly graduated RNs than places for their employment, and, as several years of nursing experience is normally a pre-requisite for entry into specialised practice this creates a delay in recruitment in areas where there are already shortages [11]. Thus, there is a need to examine the factors influencing recruitment and retention into speciality nursing practice.

RNs in Australia, similar to other countries, work as either generalists or specialists. For the purpose of this study, generalist RN practice has been defined as:

*Encompassing a comprehensive spectrum of activities*. *It is directed towards a diversity of people with different health needs*. *It takes place in a wide range of health care settings and it is reflective of a broad range of knowledge and skills*. *Generalist practice may occur at any point on a continuum from beginning to advanced* [15].

Speciality practice has been defined as a practice that follows and builds on generalist practice.

*It focuses on a specific area of nursing and is directed towards a defined population or a defined area of activity and it is reactive of depth of knowledge and relevant skills*. *Specialist practice may occur at any point on a continuum from beginning to advanced* [15, 16].

To enable a RN to move from generalist to specialist, transition programs have been developed. These programs are taken post-registration as either formal degrees or as continuing development programs. The latter may articulate into a formal degree. The programs can be taken within five years of completing a pre-registration or after some time within the workforce [17]. These programs are designed to: *“assist the newly graduated or transferred nurse/midwife to acquire further general and speciality knowledge and skills in a logical*, *sequenced supported approach to effectively transition to work expectations”* [18].

Transition programs are often delivered by employers as a way of ‘growing their own’ specialists. In the current climate of projected and real shortages, many employers have therefore designed and delivered their own programs. Thus, a transition program into peri-operative nursing, for example, can vary considerably from one employer to another as there are no national standards to regulate the content of the program.

This study was commissioned by the State Government of Queensland’s Health Department. The need for this study was a growing shortage of experience registered specialist nurses in several areas, specifically pre-operative, paediatrics, neonatal, critical care, mental health and rural and remote area nursing. To overcome the current and projected future shortages, this study sought to understand the factors that influence recruitment into and retention within these nursing specialities from both an early career (defined as within 5 years of registration) and rapid transition (after 5 years) perspectives [19]. This study was designed, therefore, to gather evidence-based data to support the rapid and early career transition of a RN from a generalist to a specialist role.

## Methods

The overall study aimed to provide an evidence-based model for early career and rapid specialisation to nursing specialities. The project contained three studies: a systematic review of the literature, interviews with RNs who had participated in a recent transition to speciality practice program and a Delphi study to confirm findings of stages 1 and 2 and to provide a model on which to base policy and education programs. The aim of study one, the systematic review, was to identify, assess and summarize available evidence relating to early or rapid career transitioning into a specialty area of nursing practice. The specialty areas considered for this review were peri-operative, mental health, neonatal, critical care, rural and remote area and emergency nursing. The primary outcome of the review was to establish a model or framework for successful early and rapid transition into speciality practice. As a secondary outcome, the review was designed to inform the healthcare and education industry of the inhibitors and enablers, influencing an effective transition into specialty areas.

### Design

This systematic review was designed and reported in accordance with international guidelines: the Preferred Reporting Items for Systematic Reviews and Meta-Analyses (PRISMA) [20]. Full details of the overall search strategy can be found in the research protocol, registered with the International Prospective Register of Systematic Reviews (PROSPERO) [19]. A convergent qualitative synthesis design, as outlined by Pluye and Hong [21], was used where results from qualitative, quantitative and mixed methods studies were transformed into qualitative findings.

### Search strategy

The search of the literature was undertaken from February to May, 2018. Articles were identified through a search of CINAHL, Medline, Scopus and PsycINFO databases. The search terms employed were nurse, early career, rapid career, transition, specialty, and Medical Subject Heading (MeSH) terms related to professional development, educational, nursing and continuing. Studies undertaken within Australia, New Zealand, United Kingdom, Canada, United States and Europe were included in the search. The results from the search were imported into EndNote X8 to remove duplicates and manage the references through the different stages of the review (full title, abstract, and full text). Titles and/or abstracts of studies were retrieved using the search strategy. Hand searching was also used to screen and identify studies that may have been missed. All papers were screened independently by two review authors to identify studies that potentially met the inclusion criteria. Inclusion criteria were: critical care, peri-operative, mental health, rural and remote, neonatal and emergency nursing, early career, rapid transition, nursing, published in English from 2007–2018. While it is recognised that nursing shortages have been a foci for several decades, the ten year time frame was selected as the shortages within nursing specialities, particularly in Australia, have accelerated, particularly influenced by the anticipated retirement of the aged workforce. Systematic or literature reviews were excluded from the study as were articles from the grey literature and research papers involving transition to general nursing (specifically new-graduate transition to generalist nursing) and all other nursing specialities.

### Risk of bias (quality) assessment

The quality of the relevant literature was appraised by two authors who independently assessed for risk of bias. Due to the lack of randomized controlled trials, and the predominance of surveys, quantitative papers were critically appraised using the Critical Appraisal Questions for Surveys (CAQS) tool [22]. Qualitative papers selected for retrieval were assessed by two independent reviewers for methodological validity prior to inclusion in the review using standardized critical appraisal instruments from the Joanna Briggs Institute Qualitative Assessment and Review Instrument (JBI-QARI) [23]. Any disagreements that arose between the reviewers were resolved through discussion, or with a third reviewer. Three qualitative manuscripts that did not demonstrate Human Research Ethical or International Review Board Clearance were excluded from the study.

**Fig 1:**
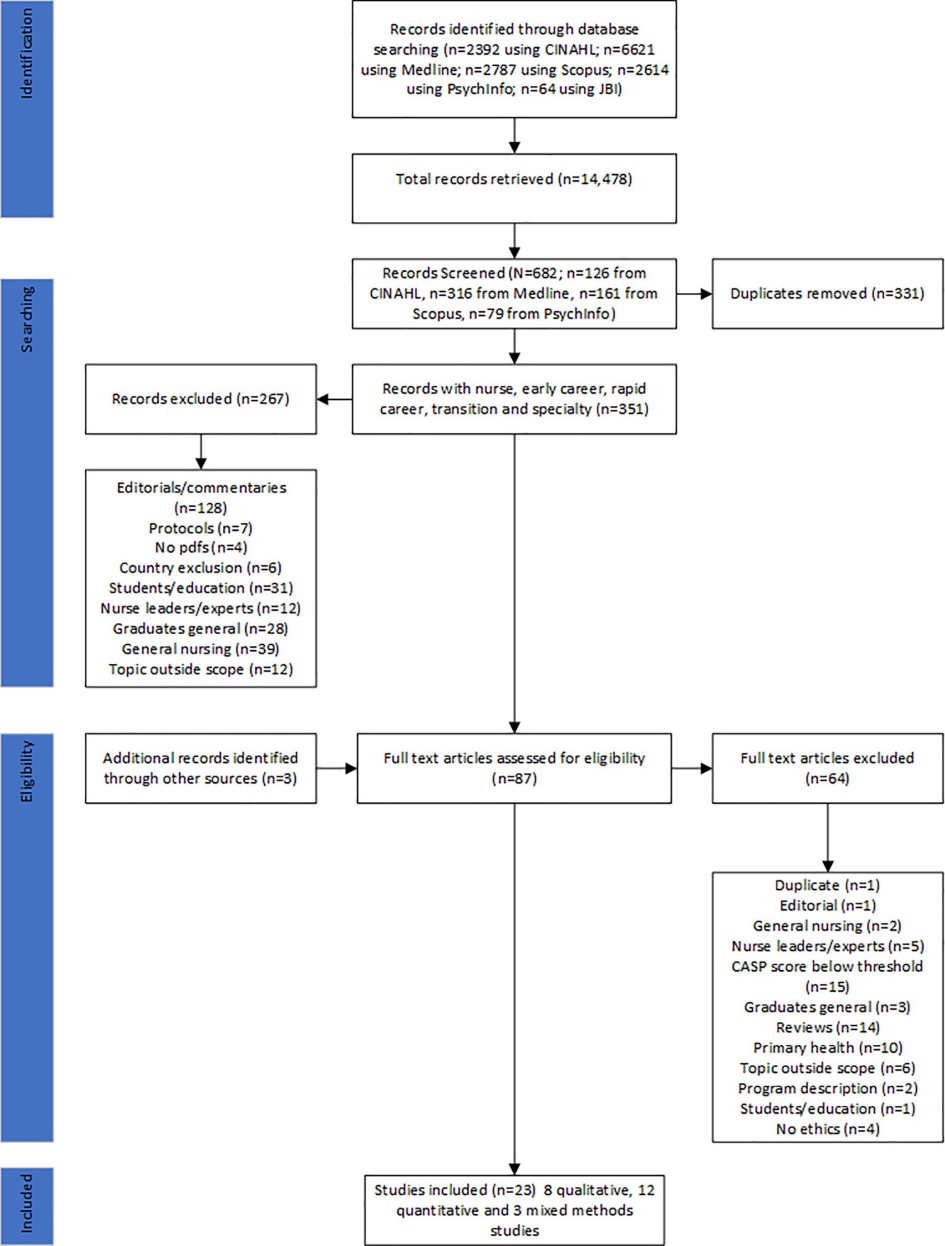
The PRISMA flowchart.

To avoid publication bias, each of these studies were assessed for overlap between sub-studies, however, none of the data appeared to repeat. Disagreements between appraisal scores were resolved through discussion and an acceptable quality appraisal. A cut-off score of 6 out of 10 for quantitative studies using the CAQS tool was adopted. Reasons for exclusion were: insufficient information on recruitment processes; measurement bias unclear; confounding factors in design and/or analysis unclear; results flawed; insufficient discussion of implications for transferability or generalizability [22]. For qualitative studies a cut-off score of 7 out of 10 was adopted. Reasons for exclusion were: lack of congruity between the research methodology and the methods used to collect the data; lack of congruity between the research methodology and the interpretation of results; the influence of the researcher on the research and vice versa were addressed; participants and their voices are adequately represented; conclusions drawn in the research report flow from the analysis, or interpretation, of the data [24]. Mixed methods studies were included if the quantitative and qualitative sub-study scored above the identified appraisal cut-off. The results of the literature search uncovered 23 articles to be included in the review (12 quantitative, 8 qualitative, 3 mixed methods) (Fig 1 and Table 1). The PRISMA flowchart (Fig 1) was used to summarise the selection process with reasons for exclusion of the study [20]. The data were analysed using matrix code analysis.

**Table 1:** Articles included in the systematic review.

Authors	Year	Title	Journal	Design	Sample Size	JBI Score	CASQ Score	Results/Findings
Boyer, S. A., Valdez-Delgado, K. K., Huss, J. L., Barker, A. J., & Mann-Salinas, E. A. [25]	2017	Impact of a nurse residency program on transition to specialty practice	Journal for Nurses in Professional Development	Quantitative Workplace Survey Tool	N = 152	N/A	8/10	There was no statistically significant difference in the total survey score pre-intervention vs post-intervention for either control or intervention group. Although not statistically significant, a small trend of positive change is apparent in the intervention group. The items rating the greatest improvement are those detailing competency development, communications, conflict management, critical thinking growth, teamwork and support for patient safety.
Bromley, P. [26]	2018	Capability: How is it recognised in student nurses undertaking postgraduate studies in neonatal intensive care?	Journal of Neonatal Nursing	Qualitative Grounded Theory	N = 4	9/10	N/A	Capability is a more precise measure and can be evaluated through various verbal and nonverbal behavioural cues. Areas used to define capability included: professionalism, interpersonal interactions and knowledge and skills.
Bromley, P. [27]	2015	Using eDelphi to identify capability requisites for postgraduate certificate in Neonatal Intensive Care Nursing	Journal of Neonatal Nursing	Qualitative Delphi	N = 25	8/10	N/A	Delphi technique used to identify capability requisites in students (qualified Registered Nurses and/or Midwives) enrolled any Postgraduate Certificate in Neonatal Intensive Care Nursing (PG Cert NICN) at any Tertiary Education Institution (TEI) in Australia. Study identified 20 themes and capability requisites that were required to be achieved at different timeframes.
Castro, E., Click, E., Douglas, S., & Friedman, I. [28]	2016	The Professionalism of Critical Care Nurse Fellows after Completion of the Critical Care Nurse Fellowship Program	Journal of Nurses in professional Development	Quantitative Electronic survey with Hall's Professionalism Inventory Scale	N = 110	N/A	7/10	46.4% of the critical care nurse fellows were in the high professionalism group and 40% in the medium professional group. Nurses with high level of professionalism rated themselves as higher on belief in self-regulation (89.7%) and sense of calling in the field (83.2%). The differences on all five subscales of professionalism on Halls Professionalism Inventory Scale (HPIS) was found to be statistically significant. Further this level of professionalism of critical care nurse fellows were found to be related to their participation in the clinical ladder advancement program.
Cleary, M., Matheson, S., & Happell, B. [29]	2009	Evaluation of a transition to practice programme for mental health nursing	Journal of Advanced Nursing	Quantitative Survey—adapted from previous research- 20 item The Nurses' Self-Concept Questionnaire—36 item	N = 45	N/A	8/10	Majority of participants (93%) were moderately to very satisfied with the overall supportiveness of nursing staff, helpfulness of other healthcare professionals, supportiveness of nursing staff, supportiveness of preceptors and clinical facilitators. Participants rated themselves as being more knowledgeable and confident post programme in all areas except information technology and interdisciplinary teamwork. No statistical differences were found pre and post responses in the Nurses' self-concept questionnaire. 91% of participants said their placement had given them an overview of mental health nursing and that they intend to continue to work in mental health nursing.
Cleary, M., Horsfall, J., Mannix, J., O'Hara‐Aarons, M., & Jackson, D. [30]	2011	Valuing teamwork: Insights from newly-registered nurses working in specialist mental health services.	International Journal of Mental Health Nursing	Qualitative Semi-structured interviews	N = 13	10/10	N/A	Qualitative study documenting the experience of mental health nurses in the first 2 years of employment. Eleven issues were identified which included: (i) teamwork; (ii) experiential learning; (iii) self-development; (iv) confidence; (v) listening; (vi) rapport; (vii) keen observation; (viii) patience; (ix) empathy; (x) learning from colleagues; and (xi) maintaining a positive approach towards patients.
Coughlan, L. M. & Patton, D. [31]	2018	A qualitative descriptive exploration of the educational and career plans of early career neonatal nurses and midwives: An Irish perspective	Nurse Education in Practice	Qualitative Descriptive qualitative approach	N = 12	9/10	N/A	An explorative study about the educational and career plans of early career neonatal nurses and midwives. Three main themes were identified which were: support and involvement, mentoring, and career progression and retention
Gillespie, B. M., Chaboyer, W., Wallis, M., & Werder, H. [32]	2011	Education and Experience Make a Difference: Results of a Predictor Study	AORN	Quantitative 98-item Revised Perceived Competence Scale (PCS-R)	N = 345	N/A	7/10	Perioperative competence was measured across 8 domains that reflect knowledge, skills and attitudes: professional knowledge, technical & procedural knowledge, practical knowledge, aesthetic knowledge, teamwork, communication, coordination, clinical leadership. Nurses with 5 years or fewer years of OR experience reported considerably lower levels of perioperative competence than their more experienced counterparts. Years of perioperative experience and specialty qualifications accounted for 23/3% of the variance in nurses' perceived perioperative competence.
Glynn, P., & Silva, S. [33]	2013	Meeting the needs of new graduates in the emergency department: a qualitative study evaluating a new graduate internship program	Journal of Emergency Nursing	Qualitative Interviews	N = 8	9/10	N/A	The study focuses on experiences of new graduate nurses in an internship program in an emergency department to help them becoming oriented into the critical care area. The study concluded that a structured program benefited participants. Preceptors were also found to be beneficial.
Gohery, P., & Meaney, T. [34]	2013	Nurses' role transition from the clinical ward environment to the critical care environment	Intensive and Critical Care Nursing	Qualitative Heideggerian phenomenology	N = 9	10/10	N/A	Study explores the experiences of nine nurses that have transitioned to the critical care environment Four main themes emerged: The highs and lows, you need support, theory—practice gap, struggling with fear. The participants felt ill prepared and inexperienced to work within the stressful and technical environment of critical care due to insufficient education and support.
Halfer, D., Graf, E., & Sullivan, C. [35]	2008	The Organizational Impact of a New Graduate Pediatric Nurse Mentoring Program	Nursing Economics	Quantitative Job satisfaction tool (investigator designed)	N = 296	N/A	6/10	Overall job satisfaction was significantly higher in the post-internship group compared to the pre-internship group. No statistically significant difference was found between job satisfaction and birth year. Statistically significant differences were found between night shift and 3 areas of job satisfaction: ability to identify work resources, ability to manage demands of job and having information to perform job effectively. Improved job satisfaction was also reflected in lower turnover rates (12% in post-implementation and 20% in pre-implementation groups).
Hussein, R., Everett, B., Hu, W., Smith, A., Thornton, A., Chang, S., & Salamonson, Y. [36]	2015	Predictors of New Graduate Nurses' Satisfaction with their Transitional Support Programme	Journal of Nursing Management	Quantitative Manchester Clinical Supervision Sale (MCSS-26) - 26 item Practice Environment Scale Australia (PES-AUS) - 30 item	N = 109	N/A	7/10	64% of the participants were allocated to work in non-critical care area, with the remaining allocated to critical-care area in their first rotation. The mean Manchester Clinical Supervision Scale (MCSS) 26 score with 73.4 (SD 11.4) and the Practice Environment Scale–Australia (PES-AUS) score was 112.3 (SD 16.2). Nurse graduate nurses (NGNs) who were younger (<23 years) were more satisfied with the practice environment compared with those who were older (p = 0.024). Three variables—unite satisfaction, satisfaction with clinical supervision and being assigned to critical-care areas were forerunners to be independent and statistically significant predictions of NGNs satisfaction. 32.5% of the variance in PES-AUS scores was explained by these.
Klingbeil, C., Schiffman, R. F., Ziebert, C., Totka, J. P., Schmitt, C. A., Doyle, L., … & Johnson, N. [37]	2016	Transition of experienced and new graduate nurses to a paediatric hospital	Journal of Nurses in Professional Development	Quantitative The Casey-Fink Graduate Nurse Experience Nurse transition difficulties and supports (investigator developed study)	N = 118	N/A	7/10	Newly hired registered nurses at a paediatric hospital were measured at 4 time points—3,6,12 & 18 months. More experienced gathered brought better communication and leadership skills over time
Krapohl, G., Manojlovich, M., Redman, R., & Zhang, L. [38]	2010	Nursing Specialty Certification and Nursing-sensitive Patient Outcomes in the Intensive Care Unit	American Journal of Critical Care	Quantitative The Conditions for Work Effectiveness Questionnaire-II	N = 866	N/A	8/10	Workplace empowerment is defined as 4 contextual factors—opportunity, information, support and resources. There was a statistically significant relationship between certification and overall perception of empowerment. The 3 patient outcome variables were: rate of central line catheter-associated blood stream infection, rate of Ventilator-Associated Pneumonia (VAP) and prevalence of pressure ulcers as likely to be sensitive to nursing practice. No statistically relationship was detected between proportion of certified nurses on a unit and patient outcomes.
Morphet, J., McKenna, L., & Considine, J. [39]	2008	The Career Development Year: Responding to the Emergency Nursing Shortage in Australia	Australasian Emergency Nursing Journal	Quantitative Self-reported questionnaire	N = 87	N/A	6/10	The majority of respondents reported having medical (76.9%) and surgical (86.5%) experience prior to commencing in the Career Development Year (CDY) program. CDY was found to have a positive impact on recruitment with 48.1% participants reporting they would not have entered emergency nursing without CY. Clinical support, orientation and study days were the three highest reasons reported for participation in CDY. Reasons that may adversely affect recruitment were: inexperience, lack of knowledge and lack of confidence. Short-term and long-term retention was found to be higher in DCY participants compared to non-CDY participants.
Morphet, J., Kent, B., Plummer, V., & Considine, J. [40]	2015	The effect of Transition to Specialty Practice Programs on Australian emergency nurses' professional development, recruitment and retention	Australasian Emergency Nursing Journal	Mixed Method Survey 1—staffing profile Survey 2—adapted from Transition to Intensive Care Nursing, Survey of Existing Education/Transition Programs and Resources' survey 13 Interviews	N = 118 ED’s N = 13	7/10	7/10	The study examines the professional development, recruitment and retention outcomes of Australian emergency nursing Transition to Specialty Practice Programs (TSPPs) TSPPs were offered in 72.1% of the ED s surveyed. Most of interviewees reported that Transition to Speciality Practice (TSPP) participants progressed more quickly than novice ED nurses who did not complete a TSPP. Two key features—development of theoretical knowledge through study days and clinical preparation and TSPP participants were employed in a group—were reported to facilitate participant professional development. Increasing recruitment and retention was the primary motivation for the introduction of TSPPs. The more years an emergency nursing TSPP had been offered, the greater the percentage of rostered nurses who have completed an emergency nursing TSPP in that ED.
Morphet, J., Plummer, V., Kent, B., & Considine, J. [41]	2017	A framework for transition to specialty practice programmes	Journal of Advanced Nursing	Mixed Method 2 surveys adapted from Transition to Intensive Care Nursing, Survey of Existing Education/Transition Programs and Resources' Survey 13 interviews	N = 118 N = 13	7/10	8/10	Duration of Transition to Specialty Programs (TSP) programmes ranged from 1 to 36 months. Emergency Departments (EDs) with programmes of 6 months’ duration had higher percentage of nurses who held a relevant postgraduate qualification and significantly higher percentage of Clinical Specialist staff than those with programmes of duration of 12 months. TSP programmes were reported to increase the rate of clinical progression among participants. Based on the results from this study; TSP programme should be for Registered Nurses with at least 1 year post registration nursing experience, participants should be employed in groups of 4–6 participants and optimal duration of programme is 6 months.
Munroe, B., Curtis, K., Murphy, M., Strachan, L., Considine, J., Hardy, J., … & Buckley, T [42]	2016	A structured framework improves clinical patient assessment and nontechnical skills of early career emergency nurses: a pre-post study using full immersion simulation	Journal of Clinical Nursing	Quantitative Pre and Post intervention evaluation	N = 38	N/A	9/10	Participant performance improved in the post evaluation test following an educational programme designed to improve nontechnical skills.
Patterson, B., Bayley, E. W., Burnell, K., & Rhoads, J. [43]	2010	Orientation to emergency nursing: perception of new graduate nurses	Journal of Emergency Nursing	Mixed Methods Interviews and surveys	N = 18	6/10	6/10	Understanding the experience of new graduate nurses to the emergency setting provides crucial information for orientation program design. Incorporating active teaching and socialization strategies early in the program may facilitate the transition from novice to beginning competent emergency nurse. Participants shared their perceptions of why they had been attracted to the program, characteristics of the emergency department and emergency nursing, being in a new job and role, reflections on their performance, the classroom and clinical components of the program, and their recommendations for future orientation programs. Results of the quantitative survey on participants’ perceptions of their first job as a registered nurse indicated that they found the work of the orientation program to be stressful.
Saghafi, F., Hardy, J., & Hillege, S. [44]	2012	New graduate nurses' experiences of interactions in the critical care unit.	Contemporary Nurse	Qualitative Descriptive phenomenology	N = 10	10/10	N/A	Perceptions of ten new graduate nurses in Intensive Care Units of their ability to interact with others. The perceptions are influenced by both how they see themselves and how they perceive that others see them.
Samedy, K., Griffin, M. T. Q., Capitulo, K. L., & Fitzpatrick, J. J. [45]	2012	Perceptions of structural empowerment: differences between nationally certified perinatal nurses and peri natal nurses who are not nationally certified	Journal of Continuing education in Nursing	Quantitative Conditions of Work Effectiveness Questionnaire- II (CWEQ-II) tool modified by Laschinger, Finegan, Shamian, and Wilk (2001) was used to measure perceptions of structural empowerment.	N = 80	N/A	8/10	Registered nurses who were nationally certified in a perinatal specialty had higher total empowerment scores and higher scores on five of the six subscales. This study supports the results of previous studies focused on differences in empowerment among nationally certified nurses and nurses without national certification. Because much of the preparation for certification is done within continuing education, nurse leaders must be cognizant of the value of certification and must develop and implement programs to support certification in the workplace.
Schwartz, L., Wright, D., & Lavoie-Tremblay, M. [46]	2011	New nurses' experience of their role within inter-professional health care teams in mental health.	Archives of Psychiatric Nursing	Qualitative semi-structured interviews	N = 10	9/10	N/A	Study explored new nurses’ experience of their role within inter-professional healthcare teams in mental health in Canada. Adopting a passive role to learn how to fit in and engaging in an active role to impact on patient care. Establishing credibility and building trust were central to the new nurses’ transition from a passive to a more active role. Interpersonal and organizational factors contributed to the transition. Recommendations for creating healthy work environments that promote interprofessional collaboration and facilitate new nurses’ transition into interprofessional health care teams are presented.
Spence, K., Sinclair, L., Morritt, M. L., & Laing, S. [47]	2016	Knowledge and learning in specialty practice	Journal of Neonatal Nursing	Quantitative Three part questionnaire	N = 208	N/A	7/10	Nurses with less than one-year speciality experience typically had the lowest Neonatal Intensive Care, Basic Knowledge Assessment Tool, Version 4 (NICU-BKAT4) scores and ‘critical’ knowledge essential for safe practice (p’s < .001). Patterns of learning emerged, with the majority (97%) of novice nurses showing a preference for experiential ‘on-the-job’ learning.

JBI = Joanna Briggs Institute; QARI = appraisal tools for qualitative studies; CASQ = Critical Appraisal Questions for Surveys; N/A = Not applicable

### Data synthesis

The quantitative and mixed method full text papers could not be used for a quantitative pooled analysis due to the diverse range of results and small sample sizes. We used a qualitative coding template thematic analysis [48] where extracts were coded into broad themes and uploaded electronically into NVIVO 11 [49] for matrix code analysis. This revision of coding produced templates that were flexibly structured as a result of emergent and unanticipated data [50] into a matrix code analysis and frequencies. This formed a framework for the model, which was confirmed and then expanded by the qualitative meta-synthesis.

Independently to the matrix code analysis and frequencies, qualitative research findings were pooled using the JBI Qualitative Assessment and Review Instrument (JBI-QARI) [23]. This involved the synthesis of findings to generate a set of statements that represented aggregation, through assembling the findings rated according to their quality, and categorizing these findings on the basis of similarity in meaning. These categories were then subjected to a meta-synthesis in order to produce a single comprehensive set of synthesized findings that were formulated into an over-arching theme, three phases and three concepts. The early findings were discussed by the quantitative and qualitative teams and from this discussion, the over-arching theme, the three phases and three concepts further emerged. The over-arching theme, phases and concepts were then plotted conceptually and from this aggregation, the TRANSPEC Model was developed which is discussed in detail in the following section.

## Results

### Overall

As described in Table 1, there were twelve quantitative papers [25, 28–30, 32, 35–39, 42, 45, 47], eight qualitative papers [26, 27, 30, 31, 33, 34, 44, 46] and two mixed method studies [40, 41, 43].

### Model development

**Fig 2:**
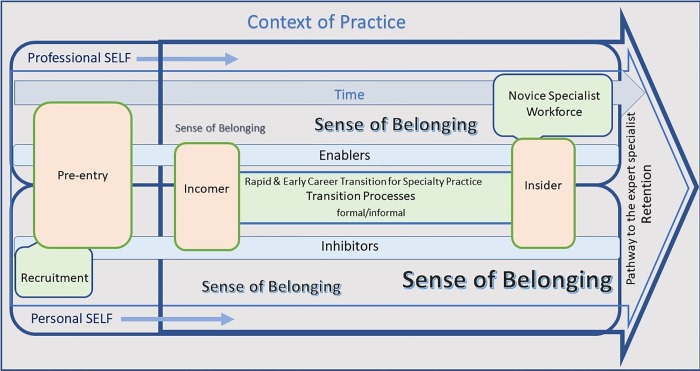
The effective early career and rapid TRANSition to a Nursing SPECiality in differing contexts of practice–the TRANSPEC model.

The thematic analysis and matrix mapping that identified the over-arching theme (context of practice), the three phases (pre-entry, incomer and insider) and three concepts (the self, transition processes and sense of belonging) were then mapped conceptually to form the TRANSPEC model (see Fig 2).

As we can see from Fig 2, central to successful transition are the concepts of the ***self*** (both personal and professional) and the development of a ***sense of belonging*** within the speciality and the context of practice. The formal and informal ***transition processes*** such as mentoring, shadowing, formal curricula, continuing professional educational short courses are a necessary part of the transition as this is the theoretical and clinical content required for speciality practice. Influencing all of this is the over-arching theme of ***context of practice*,** which can be a combination of: the geographical location; the health service type, policies and procedures; the speciality; and the links that the nurse has to other members of the multi-disciplinary team and the community in which the health service is located.

In the context of this model, the transitioning clinician goes through three distinct phases: ***pre-entry*, *incomer*** and ***insider*.** At each phase of the transition, the enablers and inhibitors influence the transition and therefore recruitment and retention. For example, pre-entry experiences such as a positive clinical placement in pre-registration programs may affect how attractive the speciality appears to the nurse and will affect steps to move into or away from the specific speciality. The TRANSPEC model (Fig 2) demonstrates the interactive nature of the overarching theme, the phases and concepts.

The findings from Tables 2, 3 and 4 are now discussed. Table 2 presents the matrix mapping which resulted in the over-arching theme and the three concepts being identified by individual papers. Within these papers, it is noted at what phase or phases these arose. Additionally, Table 2 shows the count or frequency that the over-arching theme and/or the concept or concepts were identified within each paper. Tables 3 and 4 add to the quantitative findings demonstrating the over-arching theme (Table 3) and concepts (Table 4) and findings within each phase. The enablers and inhibitors identified in each qualitative manuscript are also provided from this analysis (Tables 3 and 4). Thus the qualitative data have further informed the quantitative findings allowing for an understanding of specific inhibitors and enablers that are influencing the concepts at each phase as well the context of practice.

**Table 2:** Template and matrix coding analysis with the frequency of codes per study related to each theme and transition time point.

Authors	Year	Title	Journal	Over-arching Theme Context of Practice	Concept SELF	Concept Transition Processes	Concept Sense of Belonging	Phases
Boyer, S. A., Valdez-Delgado, K. K., Huss, J. L., Barker, A. J., & Mann-Salinas, E. A. [25]	2017	Impact of a nurse residency program on transition to specialty practice	Journal for Nurses in Professional Development	45	17	44	17	PRE-ENTRY INCOMER INSIDER
Castro, E., Click, E., Douglas, S., & Friedman, I. [28]	2016	The Professionalism of Critical Care Nurse Fellows after Completion of the Critical Care Nurse Fellowship Program	Journal of Nurses in professional Development	28	8	83	19	INCOMER INSIDER
Cleary, M., Matheson, S., & Happell, B.[29]	2009	Evaluation of a transition to practice programme for mental health nursing	Journal of Advanced Nursing	70	60	64	13	PRE-ENTRY INCOMER
Gillespie, B. M., Chaboyer, W., Wallis, M., & Werder, H. [32]	2011	Education and Experience Make a Difference: Results of a Predictor Study	AORN	105	4	141	22	PRE-ENTRY INCOMER
Halfer, D., Graf, E., & Sullivan, C. [35]	2008	The Organisational Impact of a New Graduate Pediatric Nurse Mentoring Program	Nursing Economics	70	20	74	29	PRE-ENTRY INCOMER
Hussein, R., Everett, B., Hu, W., Smith, A., Thornton, A., Chang, S., & Salamonson, Y. [36]	2015	Predictors of New Graduate Nurses' Satisfaction with their Transitional Support Programme	Journal of Nursing Management	81	19	31	29	PRE-ENTRY INCOMER
Klingbeil, C., Schiffman, R. F., Ziebert, C., Totka, J. P., Schmitt, C. A., Doyle, L., … & Johnson, N. [37]	2016	Transition of experienced and new graduate nurses to a paediatric hospital	Journal of Nurses in Professional Development	41	41	19	10	INCOMER
Krapohl, G., Manojlovich, M., Redman, R., & Zhang, L. [38]	2010	Nursing Specialty Certification and Nursing-sensitive Patient Outcomes in the Intensive Care Unit	American Journal of Critical Care	21	14	16	22	INCOMER INSIDER
Morphet, J., McKenna, L., & Considine, J.[39]	2008	The Career Development Year: Responding to the Emergency Nursing Shortage in Australia	Australasian Emergency Nursing Journal	22	120	28	10	PRE-ENTRY INSIDER
Morphet, J., Kent, B., Plummer, V., & Considine, J. [40]	2015	The effect of Transition to Specialty Practice Programs on Australian emergency nurses' professional development, recruitment and retention	Australasian Emergency Nursing Journal	47	76	32	7	PRE-ENTRY INCOMER INSIDER
Morphet, J., Plummer, V., Kent, B., & Considine, J.[41]	2017	A framework for transition to specialty practice programmes	Journal of Advanced Nursing	115	120	90	35	PRE-ENTRY INCOMER
Munroe, B., Curtis, K., Murphy, M., Strachan, L., Considine, J., Hardy, J., … & Buckley, T [42]	2016	A structured framework improves clinical patient assessment and nontechnical skills of early career emergency nurses: a pre-post study using full immersion simulation	Journal of Clinical Nursing	44	41	20	10	INCOMER
Patterson, B., Bayley, E. W., Burnell, K., & Rhoads, J. [43]	2010	Orientation to emergency nursing: perception of new graduate nurses	Journal of Emergency Nursing	106	101	16	24	PRE-ENTRY INCOMER
Samedy, K., Griffin, M. T. Q., Capitulo, K. L., & Fitzpatrick, J. J. [45]	2012	Perceptions of structural empowerment: differences between nationally certified perinatal nurses and peri natal nurses who are not nationally certified	Journal of Continuing education in Nursing	17	30	18	6	INCOMER
Spence, K., Sinclair, L., Morritt, M. L., & Laing, S. [47]	2016	Knowledge and learning in specialty practice	Journal of Neonatal Nursing	178	111	154	19	INCOMER INSIDER

**Table 3:** Context of Practice: Phases and enablers and inhibitors.

Context of Practice	Sub-themes	Enabler Findings	Inhibitor Findings
**PRE-ENTRY**	Nil	Nil	Nil
**INCOMER**			
	Trust ensuring personal safety in mental health	“…*here it's the main thing*, *trust*. *You have to trust the other… the other have to trust you because…we're a team*, *we work as a team*, *if we don't it could be dangerous here*, *anything can happen*.*”* [46] Mental Health	*Nil*
	Economic Cutbacks–causing changes to care delivery models		*“I suppose in recent times with the financial cutbacks … people haven't had the chances that they had previously” Interview 6* [31] Neonatal
	Technology–in critical care environments		*‘‘The whole ICU and HDU are very technical*. . . . . . .*even though you have very sick patients*. . . . . .*you are trying to get used to the technology*.*” (007)* [34] Critical Care
	ICT systems		*‘‘…now we have a completely different system we didn’t know where anything was and the doctors would all come in and say give me the balance for today and you wouldn’t know*.*” (006)* [34] Critical Care *‘‘Struggling with the computer system. . . .more than anything else” (004)* [34] Critical Care
**INSIDER**	Available positions		*“In the last few years [we’ve] nearly had 100% retention of our transition nurses. And the only reason now that they haven’t been staying is because we can’t give them a position” (NE 10) [40]* Emergency Department

**Table 4:** Concepts and Phases: Enablers and inhibitors.

	Sub-concepts	Enabler Findings	Inhibitor Findings
**PRE-ENTRY**			
**Self**			
***Personal***	Confidence	*“excited to learn and study” and “willing to try new things*.*”* [43] Emergency Department	*“not knowing what I don’t know” “Can I do it*?*”* [43] Emergency Department
***Professional***	Nil		
**Transition Processes**			
***Formal***	Reputation of the transition processes	*“One strategy that we really promote is that we do offer a solid education and development pathway for people that come to us*. *So that spreads and gets around*. *We’ve now got a waiting list of 15 nurses to come to us…So it has been very good for recruitment” (NUM 1)*. [40] Emergency Department	
***Informal***	Nil	*Nil*	*Nil*
**Sense of Belonging**			
***Culture***	Nil	*Nil*	*Nil*
***Working Conditions***	Pre-empting expected staff shortages		*“We have more people retiring this year than we have new staff starting*. *There's a good few people retiring and*, *even though we had interviews*, *like there's only two new staff starting*. *So we are going to be even more short staffed*. *It's going to be more pressure on everyone*. *So I think all of this should have been addressed months before now*. *For the good of the unit … maybe it should be a continuous thing that they're always doing*.*” Interview 2* [31] Neonatal
***Level of Support***	Nil		
**Context of Practice**	Nil		
**INCOMER**			
**Self**			
***Personal***	Willingness to Learn	*“…their attitude is that their inquiring mind*, *they're always stopping and asking people the questions*, *about what can I do next to improve…and you'll actually see them going out of their way to find that information” (Malcolm)* [26] Neonatal	*“the odd time something is of interest to me, I will go and pursue it. But very rarely do I go onto the databases and look for recent research.” (Interview 10) [31]* Neonatal
	Motivation/Commitment	*“I just want to be a good nurse” (RN 10)* [30] *“My career plans (are) to get more experience and be a better mental health nurse” (*RN7) [30] Mental Health	
	Fear/Anxiety		*‘‘It was a very scary experience and very frightening* …*the first day was scary and even the next few weeks” (001)* [34] Critical Care
	Being Overwhelmed		*‘‘…coming into a very busy ED*, *it puts a lot of pressure on them…how they cope*, *how they survive (NE 2)* [41]
	Conceal lack of experience		*“They would probably think ‘Oh…my god*! *You’re not very experienced’ or they might lose a bit of faith in you*. *And I probably would too if someone was looking after me and they said actually this is my first year out I would be like okay…it would be like what are they doing now … No*, *I don’t usually let on and if some just say directly how long have you been nursing…Oh well I just avoid it*. *So*, *no*, *I don’t let them know*.*” (Caroline)* [44] Critical Care
	Self-care	*“they manage to plan and structure the workload so they can actually feed themselves and water themselves and toilet themselves*.*” (Helen)* [26] Neonatal	*“they will just get their jobs done*. *They won't go to tea breaks [and will] be off late”*. *(Jenny)* [26] Neonatal
	Job satisfaction	*“I have had a couple of patients that have said*:*…‘you are a really good nurse keep going this way*, *don’t change*, *make sure you stay in nursing so that other people have the same experience that I have’; and you come out and you feel really good; and you think like wow…I really made a difference to someone*, *and maybe I did make the right decision by being a nurse*.*” (Rose)* [44] Critical Care	
	Confidence	*“Don’t be too confident*. *In the university we learn lots…but…only having the knowledge is not enough*. *You need the skill*. *You need the experience” (RN 2)* [30] Mental Health *“Confidence*. *Positive thinking and study in your spare time” (RN 1)* [30] Mental Health	*“I always say that some of these girls (sic) don't know what they don't know. They try to show that they are more confident than they actually are” (Helen) [26]*[26] Neonatal *“I thought I was confident* …*when you are left* …*one to one and it would just be you*, *you do feel…nervous about carrying out the care*.*” (009)* [34] Critical Care
	Coping		*“It is fairly easy to see when they are not coping*. *You can* …*tell a lot by their body language and their facial expression*. *They get a bit flustered*, *they get disorganised*, *some of them get a little bit loud*, *their anxiety leads to verbal overtones*, *you know sort of loudness*, *you can feel the anxiety” (Helen)* [26] Neonatal
***Professional***	Wlllingness to Learn	*“The communication to say ‘I'm feeling out of my depth here’ is actually more important to me as a teacher than someone who is…too confident” (Rosalie)*. [26] Neonatal	
	Level of Knowledge		*“I mean*, *jumping right into the ER is*, *it is hard*, *because coming right out of school you don’t know everything; you’re not going to know everything; and sometimes you’re almost expected to know more than what you actually do*.*”* [33] Emergency Department
**Transition Processes**			
***Formal***	Preparation processes embedded in the reality of practice	*“the staff are better prepared and the patients benefit from that”* (NE 2) [41]	
	Volume of knowledge	*“I know that these 10 [participants] are all going to get the same message … and the same level of support”(NM 3)* [41]	*“surprise[d] at the level of skill neonatal nurses are expected to achieve in 9 months” (#24)* [27] Neonatal
	Access to education	*“In terms of career progression we have gotten an opportunity*, *you know*, *to be sponsored to do the PGDip programme which is the neonatal intensive care programme*, *and that was paid for*, *and we did get a certain amount of study days put forward for that*.*” (Interview 3)* [31] Neonatal	*“courses are expensive*. *It is expensive to go to study days … I am self-funding my Masters*, *which you know*, *is really something you had to consider before taking it on board*. *It is something I wanted to do*, *so I made the commitment to fund that myself*.*” (Interview 11)* [31] Neonatal
	Preparation for further education	*“They get used to the review by the educators*, *they get used to meeting as a group and discussing clinical issues and at the point they go into the postgraduate program*, *the leap is not as huge (NM13)* [41]	
	Feedback on Performance	*“I think they [participants] probably don’t understand the importance of having a proper feedback process*, *but from an educator perspective that’s vital” (NE 5)* [41]	
	Quality of Processes	*“great…a good program and opportunity,” “lucky to have it” or “lucky to have gotten in.” [43]* Emergency Department *“Assessments give you confidence that they [participants] actually know what they’re doing”* (NE 12) [41]	
***Informal***	Supportive staff	*“You sort of find out who you can ask and who you can’t*, *and those more willing to help you and you keep going back to those people and asking*.*” (Linda)* [44] Critical Care	*“I think you need to be careful about who you chose to go and talk to*, *because some people might not be as encouraging as others*. *So I would say not everyone is helpful or encouraging*.*” Interview 2* [31] Neonatal
	Role Models	*‘‘Brilliant*, *there is such experienced nurses*. . . . *they are unbelievable*. . . . . .*they wouldn’t make you feel stupid*. . . .*really knowledgeable” (004)* [34] Critical Care	*“you are not encouraged to develop your skills … maybe they don't have the same formal qualifications and you are technically ahead of them in qualifications*. *And they are not comfortable with that*.*” (Interview 11)* [31] Neonatal
	Observational learning	*“… because less experience…I look a lot at what others do because I think that's the way to learn more*. *The less you talk and the more you listen*.*”* [46] Mental Health	
	Conflicting information		*‘‘Things like…a blood gas some people used to draw back with a 2 ml syringe and others used to draw back with a 5 ml and when you used a 5 ml you should be using a 2 ml and I just thought…everyone had different teaching methods” (007)* [34] Critical Care
**Sense of Belonging**			
***Culture***	Job satisfaction	*“So I think it's really important for every hospital and every organisation to keep the staff*. *The only way you keep staff is job satisfaction*. *And you don't get job satisfaction if you don't feel supported*.*” (Interview 5)* [31] Neonatal	
	Staff Personalities	*“moral and ethical values and cultural values [through] confidentiality and respect*. *She would see them “trying to be fair and equitable” (Helen)* [26] Neonatal	*‘‘headstrong*, *blunt and a little bit demeaning” (006)* [34] Critical Care
	Resource Investment	*“… if we’re going to put those resources in*, *they [participants] should be progressing quickly … “(NE 8)* [41]	
	Team support	*“I have a very good experience where excellent teamwork happens*, *and (I) feel energetic and motivated*.*” (RN 10)* [30] Mental Health	*“I think first out … yes I was very intimidated by the doctors*, *I didn’t know how to approach them and sort of things like that*, *whereas if you are a more senior level you know how to communicate to the doctors and you can get things done a lot faster*.*” (Linda)* [44] Critical Care
	Feedback on performance		*“I think*, *they [senior nurses] need to say ‘yes you have had a bad day but you did well we will see you tomorrow’ just a bit of positive feedback would do wonders … Like I think*, *I had a chat with one of the other New Grads and that was probably one of the most important things that we said we were never told that we were doing well*. *You can’t judge how well you are doing unless you get feedback; unless you are getting negative criticism all the time*, *which you seem like you are when you are a New Grad*, *there is no obvious sign that you are doing really bad*.*” (Rose)* [44] Critical Care
***Working Conditions***	Remuneration		*“… we don't make that much money*.*” (Interview 9)* [31] Neonatal
	Workload allocation	*“I think*, *you know*, *when you first start out as a nurse you want to be good at everything; you want to be spending time with your patients*, *getting to know them and their families*. *And then as time progresses you realize that you don’t have the time for that*, *because you know at the beginning they only give you a couple of patients*, *but by the end*, *you have your fill* …*”* [33] Emergency Department	
	Staffing levels		*“it is short staffed and it is a stressful career in itself*. *So I suppose people don't even consider*, *you know*, *trying to improve things a lot of the time*. *They just concentrate on getting through the day without any major mishaps*.*” (Interview 10)* [31] Neonatal
***Level of Support***	Support from colleagues	*“Be prepared to learn*, *and don’t be scared*, *because everything is a learning curve*, *and you can learn a lot from your colleagues*, *and everyone’s supportive” (RN 4)* [30] Mental Health “*Commencing participants in groups was also reported to provide peer support to and ‘comaraderie among participants … They really support each other … they become good friends and support each other through*.*’*” [40]	
	Management Support	*“I would rather my staff be talking to me than just heads down*, *bum up*, *and just struggling along”*. *(Helen)* [26] Neonatal	*“I feel like sometimes some of the managers might have favourites or they might see certain attributes in certain people and not see them in others*, *and they're more inclined to put certain people forward for study days*.*” (Interview 2)* [31] Neonatal
	Perceptions of Competence		*“even at completion of neonatal course would not assume student has understanding of complexities of being in charge of ward would allow further 12 months consolidation*.*” (#4)* [27] Neonatal
**INSIDER**			
**Self**			
***Personal***	NIL	*NIL*	*NIL*
***Professional***	Competence	*“I know one of the girls (sic) hardly had [had] a ventilated low birth weight admission because every time she was on admission she never got one*. *But when she had to do it*, *like after the course*, *she had enough knowledge that she could use her intuition and know what she had to do*. *She could prioritise her care*, *she knew what was important*, *she was able to justify why she did*, *what she did*, *when she did it*, *like her timeframe was perfect”*. *(Helen)* [26] Neonatal	*“not all NICU [student] nurses are going to be able to run a busy unpredictable NICU*. *(#22)* [27] Neonatal *“I didn't feel I was as competent as them”* [46] Mental Health
	Keeping up to date	*“I think once you are interested in the area*. *You have to have a real interest in it and a love for it*. *And then*, *you do have to have the background knowledge to back up the things that you are doing and you have to keep yourself up to date*.*” (Interview 6)* [31] Neonatal	
	Willingness to Learn	“*I think you should always have education*. *I think you should always challenge yourself*. *I think the area that we are in the challenge is to constantly learn*.*” (Interview 8)* [31] Neonatal	
	Confidence	*‘‘… made you realise that what you actually learned that you are now able to… put into practice and everything after a while of you working there everything was. . . .coming together bit by bit” (007)* [34] Critical Care	
**Transition Processes**			
***Formal***	Programs enhance successful transition	*“I find the guys on the Transition to Practice will progress a lot quicker [than novices who do not participate in a TSPP]” (NE 10)* [40] Emergency Department	
	Accreditation of Course into post-graduate programs	*“…to attract more RPL [recognition of prior learning] with XXX University*, *because it’s been shown that if you can somehow set up more RPLs you are more likely to get students to progress to postgrad [postgraduate study]*, *because it does save them a heck of a lot of money…about $2*,*500 [AUD]” (NE 5)*. [40] Emergency Department	
	Embedding learning in practice	*“On the whole I noticed that I gave a longer period of ‘learning’ for the student than the rest of the group*. *I believe*. *the student needs to be supported in the learning process*. *not be expected to be completely proficient*. *[Students] do not enter as an expert*, *rather as [a] learner*. *once they have completed their study*, *they are able to consolidate their learning*.*” (#20)* [27] Neonatal	
***Informal***	Support for continuing education	*“I do feel like I have been encouraged to push myself and get more skills*, *get more education*. *And the help is there also from an education point of view regarding college work and that*. *So it has been positive*.*” (Interview 6)* [31] Neonatal	*“Barriers to education then are obviously time constraints in the unit*. *So*, *poor staffing levels*, *high sick levels*, *and you can see the difference when you are short*. *Obviously it's understandable but you might be waiting for a long time for someone to do something with you*, *because you know you can't do it on your own*.*” (Interview 4)* [31] Neonatal
**Sense of Belonging**			
***Culture***	Positive Feedback	*“I do thrive on feedback*, *I find that really important just so I can take it on and improve my skills*. *Sometimes I will ask people and go ‘am I doing the right thing’ and different stuff*, *because I like to get feedback off people about doing stuff*.*” (Linda)* [44] Critical Care	
	Promoting professional development	*“I think when they came in first people looked at them negatively*. *As in*, *oh what are they going to say to me*? *Well it was completely not like that*. *It was the other way around*. *It was what your organisation could do for you in your progression*. *What can we do in the next twelve months*, *what's your aims*, *what's your area of interest and what is there that is available*, *and we will try to get you on those*. *So I found that very beneficial and positive*.*” (Interview 6)* [31] Neonatal	
	Team support/ Acceptance	*“At first coming from the ward*, *it is really different…the doctor situation…it is a lot easier to talk to the doctors in Intensive Care*, *because you work in a tight [relationship]…you have to be communicating well with the doctors in Intensive Care whereas on the wards it wasn’t really like that …nobody spoke to the doctors”*. *(Kylie)* [44] Critical Care *…my opinions or expertise seem to actually matter…it makes for a much more satisfying work environment…that I'm part of the team and that I do make some difference in terms of care for the patients.” [46]* Mental Health	
***Working Conditions***	Nil		
***Level of Support***	Leadership opportunities		*“You could be waiting years and years and years to get the opportunity*. *Whereas if you moved abroad*, *I think they have a lot more positions*. *They have a lot more advanced practice nurses*.*” (Interview 11)* [31] Neonatal
	Supported, valued and accepted.	*“If you are kept interested you will stay*. *If you feel like you are doing things and you are having the opportunities to get more education in areas that you are interested in*, *you will be more inclined to stay*.*” (Interview 6)* [31] Neonatal	
	Empowerment	*“felt like an emergency nurse*.*” “not having a preceptor to catch me” “comfortable*,*” “prepared*,*” and “ready to do things on my own*.*” “confident*.*”* [43] Emergency Department	

### Overarching theme: Context of practice

In this model, the term *context of practice* is used to describe the interconnected factors, experiences, and opportunities, which enable or inhibit a nurse’s progress across the continuum of speciality practice within the working environment. Therefore, the context of specialty nursing practice is framed by professional and organisational elements. The professional element is based on a core body of nursing knowledge that is continually developed and refined by practice, research and innovation. Organisational elements impacting on specialty nursing practice include the geographical location, size and capability of the health service, policies and procedures that influence the scope of practice, the community in which the health care facility is located and the diversity of health care delivered by the health service to that and other communities [15, 51–53].

The frequency of codes for the over-arching theme ‘context of practice’ ranged from 17 to 178 [45, 47]. Spence et al. [47] maintain that a context of practice underpinned by an active learning environment, strong educational processes, and contemporary engineered models of care is important for specialist practice. Further, nurses prefer to acquire specialist knowledge and skills in the practice environment and need support to optimise this learning [47].

At the pre-entry phase, there were no identified inhibitors or enablers in the literature. As an incomer, there were several inhibitors including: economic cutbacks that limited the scope of experience [31] and the information and communications technology (ICT) environment [34], which were seen as a major challenge to care delivery in a new context. Specifically, nurses noted how they struggled with the technology and different ICT systems. This was particularly the case in critical care environments. One enabler at the incomer phase was the presence of trust of the incomer [46], and this was particularly important in the mental health environment. As an insider, the major influence, seen as an inhibitor was the lack of positions to retain nurses who had completed their transition process/program [40].

### Concept one: The self

The concept of ‘the self’ is seen as a complex system steeped in philosophical, psychological and sociological arguments. Thagard [54: 2] argues that the self is:

*A multilevel system consisting of social, individual, neural and molecular mechanisms… Each of these levels can be understood as a sub-system consisting of environmental influences, component parts, interconnections between parts, and regular changes in the properties and relations of the parts*.

Thagard [54] goes on to note that each of these levels are influenced by additional phenomena such as ‘self-presentation, self-esteem, self-enhancement; self-regulation; self-expansion and self-development’. For the purpose of this study the concept of ‘the self’ is defined as an individual who is constantly changing and adjusting. These changes are driven by self-preservation, self-esteem, self-enhancement, self-regulation, self-expansion and self-development at two levels. For the professional nurse, these can be described as changes to the personal and the professional self.

Frequency of the codes for the concept ‘the self’ ranged from four [32] to 120 [39, 41]. As reported by Morphet et al. [41], enhancement of the personal and professional self occurs by the use of an evidence-based transition to speciality practice framework to guide the structure, process and outcomes of speciality practice. Morphet et al. [39] reported that a dedicated “career development year” enriches the development of the personal and professional self and translates into greater recruitment and retention of speciality nurses.

#### Personal self

At the pre-entry phase, confidence was seen as both an enabler and an inhibitor [43], while at the incomer stage willingness to learn [31], self-care [26], and confidence [26, 30, 34] were seen as both inhibitors and enablers. Coping [26], being overwhelmed by the environment [41] and concealing lack of experience in the speciality [44] were inhibitors. In contrast, motivation and commitment [30] and job satisfaction [44] were seen as enablers. There were no enablers or inhibitors identified for the personal self at the insider phase.

#### Professional self

There were no enablers or inhibitors identified in the pre-entry phase. At the incomer phase, willingness to learn [26] was an enabler and the level of knowledge was seen as an inhibitor [33]. At the insider phase, competence [26, 27, 34] was seen as both an inhibitor and enabler. Keeping up to date [31], willingness to learn [31] and confidence [34] were seen as enablers.

### Concept two: Transition processes

For the purpose of this paper, transition processes refer to the processes or period of changing from one phase to another. The time to complete the transition processes will vary from one nurse to another as will the transition processes they undertake. Influences will include the context of practice, the beginning level knowledge and skills, the nature of the processes being undertaken and the availability of support [55]. Transition processes can be formal and/or informal. In some instances, both formal and informal processes were used, and in other cases only one was used.

#### Formal processes

Formal transition processes are defined as processes that involve educational and clinical support and are designed to facilitate the transition [56]. The inhibitors and enablers identified related to these formal processes and the nurse’s ability to access them.

This concept had the highest frequencies of quantitative matrix coding as most of extracts described outcomes from transition programs or educational interventions. In the highest matrix coding frequency extract, 154 codes [47], found that nurses with less than one year speciality experience typically had the lowest ‘critical’ knowledge essential for safe practice, with the majority of novice nurses showing a preference for experiential ‘on-the-job’ learning. Gillespie’s [32] paper with a matrix coding frequency of 141, reported that although both years of experience and specialty education contributed to nurses’ perceived competence, experience was the strongest contributor.

The transition processes [40] were regarded as an enabler at the pre-entry phase. At the incomer phase, preparation processes embedded in the reality of practice, the way the program prepared the nurse for further education and feedback on performance were identified as enablers [41]. Additionally, the quality of the processes was also seen as an enabler [41, 43]. The volume of knowledge to be learnt [27, 41] and the ability to access the transition processes [31] were both enablers and inhibitors. Insider enablers were the way that they processes had enhanced successful transition [40], the accreditation of the processes into post-graduate programs [40], embedding learning into practice [27] were all enablers. There were no inhibitors found at the insider phase.

#### Informal processes

No inhibitors or enablers were found at the pre-entry phase. For insiders, supportive staff [31, 44] and role models [31, 34] were both enablers and inhibitors. Observational learning [46] was an enabler and conflicting information was an inhibitor [34]. Support for continuing professional education was both an enabler and inhibitor [31] at the incomer phase.

### Concept three: Sense of belonging

Baumeister and Leary [57] define a sense of belonging as the degree to which an individual perceives as being accepted, included and supported by others in their environment. When people feel they belong, positive emotional and cognitive outcomes occur [58]. A sense of belonging can be experienced in many contexts [59]. It relates to the inclusiveness of all participants where a culture of connection is built through relationships to promote a sense of belonging [60]. The within this concept three sub-concepts emerged as important to a successful transition. These were: were the culture of the organisation, the working conditions and the level of support. The over-arching theme of the context of practice, also influenced this concept and the enablers and inhibitors within it.

#### Culture

Culture is defined as the totality of the relationships formed within the culture, and shapes how those relationships are structured [61]. The highest frequency of matrix codes was found in Morphet et al. [41], 35 codes [41], followed by Halfer [35] and Hussein [36], both 29 codes. From these studies, it was reported that speciality graduates are eager to establish goals for lifelong learning, specialty-nursing certification, postgraduate study, clinical ladder advancement, clinical governance participation, and professional organizational membership [35]. Also, it is imperative for nursing management to create opportunities for new speciality nurses and more experienced staff to develop closer working relationships, and improve retention through team projects and other department ‘bonding’ activities as these engender a sense of ‘belongingness’ among the new speciality nurses [35]. There were no identified inhibitors or enablers at the pre-entry phase. As an incomer, job satisfaction [31] and resource investment [41] were enablers while feedback on performance was an inhibitor [44]. Staff personalities [26, 34] and team support [30, 44] were both inhibitors and enablers. The culture of the organisation at the insider phase was enabled by positive feedback [44] team support/acceptance [44, 46] and promoting professional development [31].

#### Working conditions

Nurses, in the majority, are in paid work. Working conditions for paid work normally are considered to include working time (hours of work, rest periods and work schedules), remuneration and the physical conditions and mental demands that exist in the workplace. Working conditions for the purpose of this study include: skill-mix, staffing ratios, remuneration, role clarity, support, sufficient staff, workload allocation and role clarity. There were no matrix codings for this sub-concept. In the pre-entry phase the inability of organisation to pre-empt staff shortages was identified [31]. At the incomer phase, remuneration and staffing levels [31] were inhibitors, whereas workload allocation was an enabler [33]. There were no inhibitors or enablers identified at the insider phase.

#### Level of support

Support, for the purpose of this study, was defined as support within the workplace and was provided by colleagues and management. There were no matrix codes for this sub-concept. At the pre-entry phase there were no enablers or inhibitors. At the incomer phase, support from colleagues [30, 41] was an enabler, while perceptions of competent management of a ward or unit [27] was an inhibitor. Management support for their role [26, 31] was both an inhibitor and an enabler. At the insider phase, the opportunities to become a leader [31] were seen as an inhibitor and being supported, valued and accepted [31] and empowered [43] were enablers.

## Discussion

The main concepts identified in the transition process included: the Self (personal and professional), the Transition Processes (formal and informal), and a Sense of Belonging. The data also clearly identified three phases to the transition: Pre-entry, Incomer and Insider. Influencing successful transition at each phase and within each theme were the identified enablers and inhibitors. It was apparent from the data analysis that at each phase, the three major concepts of the Self, Transition Processes and the Sense of Belonging occurred. It was possible therefore to map these conceptually (hence TRANSPEC model emerged). It was also apparent that the over-arching theme of the Context of Practice, particularly the geographical location and the speciality, influenced the transition. Thus it was possible to enclose the phases and concepts within an over-arching theme of the Context of Practice. To inform the development of a transition program, it was possible also to identify enablers, inhibitors or both in each phase and concept. Thus the model developed from the data analysis.

### Context of practice

The model acknowledges the importance of the context of practice in both early career and rapid transition to specialty practice. There are many ways that the context can impact on the scope of practice on a nurse and therefore the level of professional and personal experience, skills, knowledge and competence required for practice. For example, geographical location of the health service has been found previously to influence: a) access to health services; and b) the scope of practice of the nurse [62]. Additionally, while there were many similarities in the influence of the context of practice across specialities, there were also differences that needed to be taken into consideration. For example, mental health specialities saw the need for personal and team safety [46], whereas nurses working in the critical care environment highlighted the complexity of practice caused by the technology used [34]. A third major influence on the context of practice, which has been well documented previously, are the organisational influences (e.g. funding, scope of practice restrictions, management styles) [63].

The TRANSPEC model acknowledges the importance of context of practice and allows for each speciality within a discipline to identify specific enablers, inhibitors or both that limit recruitment and then retention. It therefore allows for the different contexts of speciality practice.

### The self (professional and personal)

#### The professional self

Nationally and internationally, speciality practice has been described in terms of the movement from novice to expert [15, 16, 56, 64]. Benner’s model in particular has provided clear guidance for what is needed to advance professional practice. However, to date research has focused on specific specialities, for example, critical care, emergency department, mental health, neonatal, paediatric, rural and remote and so on. This has resulted in research and standards of practice for specialities being developed in silos. Additionally, transition to speciality practice research has focused on nurses at the incomer and insider phase. The model demonstrates that entry to professional speciality practice begins when the speciality practice is being considered as a career path (pre-entry). The importance of experience during pre-registration clinical placements and its impact on recruitment into speciality practice has been highlighted by this review [40, 65]. This review and one recent study demonstrate the importance of enablers and inhibitors at pre-entry. Yet, these have received limited attention in research [66].

#### The personal self

The findings demonstrate how the personal self is influenced by confidence, anxiety about a new working environment and coping within it [26, 34, 43]. Attention to self-care was seen as an important way to cope with the new environment [26]. Previous research and policies have focused on building the competence (skills and knowledge) of the nurse as they transition within that speciality [56]. Recent work highlights the importance of individual well-being of nurses and how factors such as burnout and resilience can not only influence retention within the workforce, but also the quality of patient care delivered [67].

### Transition processes (formal and informal)

Nursing has been acknowledged as a practice profession, with a combined theoretical knowledge (science of nursing) and the practice (art of nursing), being equally important [68]. Experiential learning, where the application of the theoretical knowledge informs practice, is important, with most health-related professions acknowledging the importance of on-the-job-training, mentorship and ongoing learning [69]. Life-long-learning (LLL) has become an important element of nursing practice through a requirement by AHPRA that all registered health practitioners provide evidence of continuing practice development, stipulating the annual minimum hours required for the continuance of registration [70]. LLL is a combination of self-directed learning, organisational learning through in-service sessions, required competence sign-offs, and required skills and knowledge acquisition in accordance with the profession or practice [71]. It requires a practitioner to take control of their learning, to engage with their profession and to actively support quality improvement initiatives that support personal and service adaptation and flexibility through evidence-based practice [71]. When a practitioner is transitioning to practice, whether it be a new area of practice, or gaining experiential knowledge as a new practitioner, both the practitioner and the service employing them have an obligation to access and provide learning opportunities within the field of practice the employment occurs. These can be divided into formal and informal processes.

#### Formal processes

The formal curricula, on the whole, began when the nurse started in the new role. Few curricula focus on developing the nurse before they enter the speciality–in the recruitment or pre-entry phase. Procter et al. [65] note the importance of a positive experience during entry-to-practice clinical placements as this experience has the potential to influence how attractive the student may see the speciality in the longer term.

Formal processes include targeted educational programs that are offered at tertiary institutions, and may be at Certificate, Diploma, Bachelor or higher degree level. For specialty practice areas, these are general postgraduate qualifications that build on existing general practice knowledge to support transition to speciality practice [40, 56]. An important feature identified was that of processes of being embedded in the reality of practice [43, 65]. The importance of the preceptor and/or mentor was emphasised with other studies into transition, for example Hooper et al. [72] noted that they were important to assist with building and maintaining resilience and self-development. Conversely, the quality and level of support from preceptors and mentors can vary and thus impede the transition process [73].

#### Informal processes

Informal processes noted in this study, employed during the transition processes included: role modelling which could be an enabler [34] or an inhibitor [31]; observational learning [46] and general supportive staff (nurses or other health care professionals) [44]. Similar to pre-entry exposure, the behaviour of staff already established in the area could be either an enabler or an inhibitor depending upon the characteristics of the individual staff member [31, 44]. Importantly, encouragement to pursue further education in the speciality was seen as an enabler [31].

### Sense of belonging

The TRANSPEC model emphasises the importance of the development of a sense of belonging within the speciality, within the health service, within the multidisciplinary team and within the community. Hospitals, just like schools and other organizations do not exist in a vacuum. They respond to the social contexts in which they are immersed. Opportunities for participation, respect for and from participants in the work as well as general community as well as being valued and accepted, can aid in realising an individual’s psychological need to belong. Such support provides a sense of belonging and a connectedness, in which social and cultural facets shape the individual and community identity [58]. When people feel they belong, positive emotional and cognitive outcomes occur [58]. There were three sub-concepts that impacted on the sense of belonging: the culture, the working conditions and the level of support provided. Additionally, was the over-arching theme of the context of the specialisation. The personalities of current employees within the organisation and their interaction with the new nurse were both inhibitors and enablers. The culture of the workplace, particularly leadership, has been shown in many previous studies to impact on transition entry to practice as well as retention within practice [74]. The importance of other staff in the transition processes have also been highlighted by previous studies noting that promotion of a working environment that encouraged ‘inter-professional collaboration’ [72] and was seen to be an enabler, while ‘maltreatment, abuse and neglect’ were seen to be inhibitors [72]. In line with these findings, this study has highlighted the importance of positive feedback and team support and acceptance [31, 44]. This is at the core of a sense of belonging as this increases mutual trust and therefore respect, wellbeing and self-esteem. The impact of working conditions such as remuneration, workload allocation, staffing levels and lack of role clarity [75] have been raised previously as well as in this study [31, 33, 72, 73]. The impact of workload and the working environment on the personal self has also been documented previously [76, 77]. The time spent in the speciality is also seen as an enabler with insiders having a successful transition if allowed time to do so [73].

Support from colleagues and management also impacted on the sense of belonging. Leadership was demonstrated by either example [26] or by opportunities [31]. Successful leadership within healthcare organisations highlights the importance of emotional intelligence for insight into the “self’ of the nurse transitioning through change [78]. Emotional intelligence involves skills that are learnt and enhanced. Developing a sense of belonging and competence in the speciality happens over time, and depends on the individual (self).

### Limitations

The review was limited to the specialities of critical care, mental health, neonatal, peri-operative and it is acknowledged that the inhibitors and enablers outlined in this review may vary from one speciality to another. However, the findings of this study are similar to those undertaken in a parallel transition to community and midwifery practice systematic review which has further developed this model for that specific content [79] and suggests that the TRANSPEC model can be applied to other nursing and non-nursing speciality contexts.

## Conclusions

The TRANSPEC model provides a foundation for effective recruitment and retention for both early career and rapid transitions to speciality practice. It shows that transition begins prior to entry to the speciality (pre-entry) and then continues to be developed over time. A successful transition relies on, at each phase, enablers to the professional and personal self, the formal and informal transition processes and a sense of belonging developing. This transition is influenced by the context of practice and the transition time will vary from one nurse to another.

The TRANSPEC model brings new knowledge to speciality transition in that it acknowledges the importance of the pre-entry phase, which has not been a focus previously. It also acknowledges that recruitment and retention into speciality nursing practice involves both the professional and personal self. The importance of personal well-being is increasingly being recognised, particularly its effects on quality patient care and patient safety.

The TRANSPEC model is designed to be used as a basis from which inhibitors and enablers can be considered. While this review has outlined some inhibitors and enablers, and recognising that there will be many of these that are not unique to a particular speciality, it is recommended that when developing a transition program using this model, the focus should be on enablers at all three phases for each individual speciality.

### Recommendations

Further testing of the TRANSPEC model should occur to ascertain its applicability to successful rapid and early career transition to a nursing speciality.

## Supporting information

S1 Fig PRISMA 2009 checklist. (DOC)
